# Innate Immune Activation and Subversion of Mammalian Functions by *Leishmania* Lipophosphoglycan

**DOI:** 10.1155/2012/165126

**Published:** 2012-02-22

**Authors:** Luis H. Franco, Stephen M. Beverley, Dario S. Zamboni

**Affiliations:** ^1^Department of Cell Biology, School of Medicine of Ribeirão Preto, University of São Paulo, FMRP/USP, 14049-900, Ribeirão Preto, SP, Brazil; ^2^Department of Molecular Microbiology, Washington University School of Medicine, 660 S. Euclid Avenue, St. Louis, MO 63110, USA

## Abstract

*Leishmania* promastigotes express several prominent glycoconjugates, either secreted or anchored to the parasite surface. Of these lipophosphoglycan (LPG) is the most abundant, and along with other phosphoglycan-bearing molecules, plays important roles in parasite infectivity and pathogenesis in both the sand fly and the mammalian host. Besides its contribution for parasite survival in the sand fly vector, LPG is important for modulation the host immune responses to favor the establishment of mammalian infection. This review will summarize the current knowledge regarding the role of LPG in *Leishmania* infectivity, focusing on the interaction of LPG and innate immune cells and in the subversion of mammalian functions by this molecule.

## 1. Introduction: *Leishmania* and Lipophosphoglycan

Leishmaniasis is caused by infection with protozoan parasites of the Trypanosomatid genus *Leishmania*. The disease is endemic in several regions, including west Asia, Africa, and South America. In humans, several disease manifestations have been observed, ranging from self-healing cutaneous lesions to progressive and fatal systemic infection [1]. Leishmaniasis is transmitted by the bite of phlebotomine sand flies and in most parts of the world is a zoonosis, although in some areas direct human-fly-human transmission has been reported [1].

The life cycle of *Leishmania* has two main morphological forms: flagellated promastigotes, which replicate and develop in the midgut of the sand fly vector, and rounded amastigotes, which live and multiply inside the macrophages of the vertebrate host. The establishment of the infection begins with the inoculation by the sand fly vector's bite of metacyclic promastigotes into the vertebrate host. From this wound site, the parasites encounter a variety of cell types including neutrophils, Langerhans and dendritic cells, keratinocytes, and tissue macrophages, all of which have been proposed to serve as the “first contact” host cell (reviewed in [2]). While *in vitro* and in some cases *in vivo* studies provide good support for these models, the complex nature of the sand fly bite makes it difficult to ascertain the quantitative importance of these to the final parasitic outcome. Ultimately, the metacyclic forms of the parasite are internalized and differentiate intracellularly to the amastigote form. In macrophages, amastigotes multiply inside the acidic vacuoles, and eventually are released after lysis, spreading the infection to uninfected cells [3]. Current knowledge about the steps leading to parasite escape is limited, for example, whether it is regulated by the parasite or occurs simply through overwhelming the capacity of the macrophage to harbor them.

*Leishmania *promastigotes are covered by a thick glycocalyx comprised of abundant glycoconjugates important for parasite survival and pathogenesis. These molecules include Lipophosphoglycan (LPG), proteophosphoglycan (PPG), gp63 metalloproteinase, and glycophosphatidylinositol lipids (GIPLs). One notable feature distinguishing the *Leishmania* surface from that of the host is that most parasite molecules are linked to the parasite surface through glycosylphosphatidylinositol (GPI) lipid anchors [4–8].* Leishmania* also secrete protein-linked phosphoglycans (PGs), such as the secreted proteophosphoglycan (sPPG) and secreted acid phosphatase (sAP) [9].

**Figure 1:**
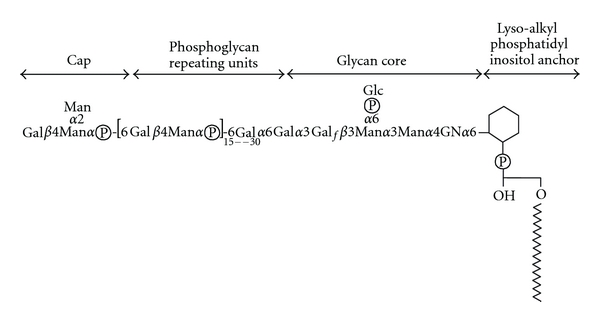
Structure of Lipophosphoglycan from *Leishmania donovani*. The four key domains (cap, phosphoglycan repeating units, glycan core and lipid anchor) are discussed further in the text. The number of phosphoglycan (PG) repeating units increases during metacyclogenesis, contributing to the role of LPG in complement resistance. In many *Leishmania *species, side chain modifications of the PG Gal residue are common, where they can play a role in sand fly transmission. The structure of the cap also differs amongst species. Gal, galactose; Man; Mannose; GN, glucosamine; Glc, glucose.

LPG is the most abundant glycoconjugate on the surface of *Leishmania *promastigotes. The GPI anchor which links LPG at surface of the parasite is constituted by a 1-*O*-alkyl-2-*lyso*-phosphatidyl(*myo*)inositol lipid anchor with a heptasaccharide glycan core, to which is joined a long PG polymer composed of 15–30 [6-Gal(*β*1,4)Man(*α*1)-PO_4_−] repeating units, and terminated by a capping oligosaccharide (Figure 1). The PG repeating units are often modified by other sugars, which are typically species and stage specific. Procyclic and metacyclic promastigotes of all *Leishmania* species express high amounts of LPG on their surface, in contrast to amastigotes, whose LPG expression is highly downregulated [10]. In promastigotes, LPG plays an important role for parasite survival inside sand fly vector and for macrophage infection, as discussed below. In contrast, the survival of amastigotes inside host macrophages is improved by other PG-containing glycoconjugates, such as PPG, which are highly expressed on its surface. All of the LPG domains are shared with other parasite surface molecules, to varying extents and degrees of relatedness. The PG repeat, side chains, and caps can be found on PPG or sAP, and both the GPI glycan core and lipid anchor have similarities with those present in both GIPLs and GPI-anchored proteins [8, 11, 12]. As described below, the usual of mutants defective in specific steps of LPG biosynthesis have proven useful in resolving the role of LPG domains clearly from related ones borne by other molecules. 

## 2. The Role(s) of LPG and PGs in the Sand Fly Vector

**Figure 2:**
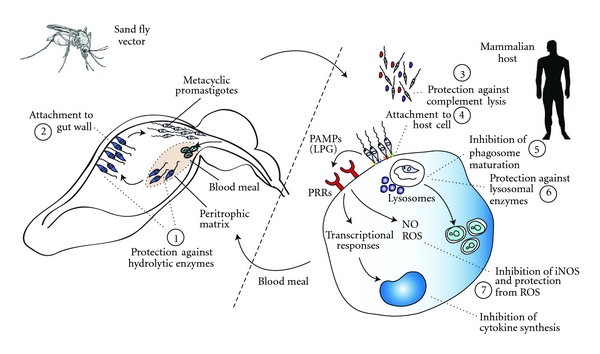
Role of LPG in *Leishmania* infectivity and virulence. Shown are putative and bona fide actions of *Leishmania* spp. LPG molecules in subversion of host and vector functions. These LPG functions include (1) physical protection to promastigotes against hydrolytic enzymes in the digestive tract of insect; (2) attachment of promastigotes to the gut wall; (3) In the mammalian host, promastigotes protection against lysis by complement proteins; (4) attachment of parasites to the macrophage membranes or alternative transiently infected cells, such as neutrophils, dendritic cells and perhaps others; (5) transient impairment of the phagosome maturation; (6) physical protection against degradation by lysosomal enzymes; (7) modulation of macrophages activation through impairing the synthesis of nitrogen species and cytokines related to the control of infection and protection from ROS.

A number of obstacles present in the sand fly vector digestive tract are potentially able to impair the development of *Leishmania*, including digestive enzymes, the midgut peritrophic membrane barrier, avoidance of excretion along with the digested blood meal, and the anatomy and physiology of the anterior gut (Figure 2). These barriers have provided the evolutionary drive for expression of molecules by the parasite required for successful development in the sand fly vector. As in the mammalian stages emphasized in later sections, LPG and related PGs are key molecules important for survival inside the hostile environment of sand fly vector [9]. 

During the digestion of blood meal in the insect midgut, the intracellular amastigotes initiate their differentiation to the motile procyclic promastigotes. These forms of the parasite leave the macrophages and are exposed to the hostile environment of the midgut. The dense glycocalyx formed by LPG and PPG provides protection against the action of midgut hydrolytic enzymes and by inhibiting the release of midgut proteases [13]. Procyclic promastigotes are able to attach to midgut epithelial cells, which enable the parasite to be retained within the gut during excretion of the digested blood meal. Several findings have suggested that LPG plays an important role in attachment of promastigotes in midgut in some species or strains such as the *L. major* Friedlin line [14–16], which binds to the sand fly midgut lectin PpGalec [17]. However, in other species, LPG appears to play less of a role in attachment, as LPG-deficient mutants retain the ability to bind [18, 19]. The molecules mediating this attachment are unknown although a role for parasite lectins has been suggested [20, 21]. For those strains/species dependent upon LPG for binding, the parasite must then find a way to release from the midgut in order to be free for subsequent transmission. To do this, metacyclic parasites synthesize an LPG unable to interact with host lectins. For *L. major *strain Friedlin, the procyclic Gal-*β* 1–3 PGs of LPG are“capped” with D-arabinopyranose, resulting in an LPG unable to bind PpGalec [17, 22]. In contrast, in *L. donovani* which synthesizes an LPG lacking PG modifications, binding through the terminal capping sugar is “masked” through elongation of the LPG chain [23].

The promastigote stage of many *Leishmania* species elaborates a thick mucoid “plug” during infections, comprised primarily of PPGs along with other shed parasite molecules. At the time of transmission by biting, the plug contents are inoculated along with parasites and saliva into the host. Seminal studies by Bates and collaborators have suggested that the PG repeats borne on PPGs within the plug play key roles in exacerbating the subsequent infections in *L. mexicana*, thereby implicating PGs synthesized and secreted by *Leishmania *in the fly as important immunomodulators of the host response [24, 25]. Notably sand fly saliva can exacerbate *Leishmania *infections as well. It is worth pointing out that most experimental studies of *Leishmania* transmission are compromised to some extent by the use of needle inoculated parasites, lacking these key biological mediators as well as differing in the amount of local tissue damage.

## 3. The Role(s) of LPG and Related PGs in Mammalian Infectivity

As seen with the sand fly stages, LPG and related PGs have been implicated in a variety of key steps required for infectivity of mammalian hosts (Figure 2). Here, we summarize the current information regarding the role of LPG for subversion of mammalian protective responses by the parasite, and the recognition of parasite LPG by the mammalian innate immune cells.

## 4. The Role of LPG for Avoidance of Lysis by Complement

Before the internalization by host cells, metacyclic promastigotes must evade lysis by the mammalian complement system. Several studies using purified LPG or LPG-deficient parasites have shown that this molecule defends against complement-mediated lysis [26, 27].* L. major *metacyclic promastigotes, the infective forms for mammals, are resistant to complement-mediated lysis while the procyclic forms, which reside inside the sand fly vector, are highly susceptible [28]. This difference is conferred by changes in the length of the metacyclic LPG PG polymer domain, which bears about twice as many repeating units as the procyclic promastigotes. This prevents the attachment of complement membrane attack complex (MAC) and pore formation on parasite surface [28]. However, earlier steps in the complement cascade may contribute in the entrance of *Leishmania* into macrophages through complement receptors. LPG, together with the protease gp63, is able to activate the complement system, leading to the generation of the C3b and C3bi opsonins. C3b and C3bi thus bind to *Leishmania* surface and mediate the parasite phagocytosis by complement receptor (CR) 1 and CR3 [29–35]. Phagocytosis of* Leishmania *via CR1 and CR3 receptors is considered as a means of “silent entry” into macrophages, because it does not prompt the oxidative burst and impairs the production of IL-12 [32, 36–38]. However, infections of CR3-deficient mice show very little attenuation of infection, suggesting that this step may be of lesser importance or redundant with other binding interactions in survival [39].

## 5. The Role of LPG in Parasite Invasion and Survival in Macrophages

After being inoculated into the mammalian host by the sand fly vector, the metacyclic promastigotes are internalized through interactions with a number of different receptors. While at various times interactions with one or another of these have been presumed or shown to be dominant in cellular or biochemical tests, genetic studies have typically led to conclusions that these interactions typically may instead be highly redundant in biological settings. In this scenario, the use of multiple receptors allows the promastigotes to be quickly internalized by macrophages (reviewed in [40]).

Importantly, the LPG plays an important role as a ligand during the attachment and invasion process of macrophages, either directly or indirectly through binding to other proteins.One example is the interaction of LPG with mannose-fucose receptor expressed by macrophages [41]. In addition, mannan-binding protein (MBP) is able to bind to mannose residues on LPG, enabling the formation of C3 convertase and generation of C3b, which helps promastigotes to attach to the macrophage as noted above [42]. C-reactive protein (CRP) binds to LPG of *L. donovani *metacyclic promastigotes triggering their phagocytosis by human macrophages via CRP receptor [43]. Commonly, the engagement of CRP receptor by its ligand leads to macrophage activation, resulting in proinflammatory cytokine production [44, 45]. However, phagocytosis of *L. donovani* by CRP receptor leads to an incomplete activation of macrophages, thus favoring parasite replication [46].

Following entry, promastigotes are contained in a phagosome known as parasitophorous vacuole (PV), which undergoes several fusion processes, giving rise to a phagolysosome-like organelle [47, 48]. During this process, LPG acts to delay PV fusion with lysosomes, promoting delay in PV acidification and acquisition of lysosomal enzymes [49]. Vacuoles harboring promastigotes of *L. donovani* and *L. major* genetically deficient for LPG fuse more extensively and rapidly with endosomes and lysosomes [27, 50]. While initially workers postulated that this delay protected promastigotes from acidic conditions and hydrolytic enzymes until they had differentiated to the more acidophilic amastigote stage, work with LPG-null* L. major *promastigotes provided little support for this model [27], as these parasites are able to survive under several conditions despite rapid fusion with host lysosomes. Instead, the delay in fusion reflects changes in membrane properties that result in delocalization of the host oxidative burst from its normal peri-PV location [51]. On top of this, LPG itself is able to interact and deflect oxidants directly [52]. Other roles of the delayed fusion may not only concern survival but immune recognition and antigen processing, which is dependent on host hydrolytic enzymes [50].

Whereas LPG seems to be important to protect *Leishmania* during differentiation from promastigote to amastigote forms, it does not play a significant role during the development of the amastigote form. Indeed, LPG expression on amastigotes of several species of *Leishmania* is highly downregulated (1000 fold or more) [10] suggesting that the protective role of LPG is transient and limited to the beginning of host cell infection. However, other PG-containing glycoconjugates and especially PPG are expressed at high levels in amastigotes, and act in PG-dependent manner to protect the amastigote [53, 54].

## 6. The Role of LPG for Inhibition of Macrophage Activation

Infected macrophages employ several microbicidal mechanisms to eliminate intracellular pathogens. When previously activated by interferon-gamma (IFN-*γ*) and tumor necrosis factor-*α* (TNF-*α*) or other microbial components, infected macrophages express high levels of the inducible nitric oxide synthase (NOS2), culminating with production of nitric oxide (NO), NO_2_^−^, and NO_3_^−^ [55]. These nitrogen intermediates coordinate processes that lead to deprivation of important components, such as iron, which lead to restriction of intracellular parasites replication [56]. *L. major* is able to induce higher amounts of NOS2 in the cutaneous lesion and draining lymph nodes of the clinically resistant lineage C57BL/6 compared to the nonhealing BALB/c strain [57]. In addition, mice deficient for NOS2 are more susceptible to infection with *Leishmania*, compared to their littermate controls, as well as macrophages derived from these mice [58–60]. Thus, the production of NO is indispensable for the control of *L. major* infection and for maintaining life-long control of persisting *Leishmania* parasites [61–63].

In contrast, infection of unactivated macrophages typically leads to parasite survival and minimal levels of NOS2-dependent NO production, to the point that *L. major* was referred to as a “stealthy parasite” [64]. Thus, one of the challenges in experimental models is the need to distinguish infections where macrophages are naturally or experimentally activated from those situations where *Leishmania* exhibits successful parasitism and survival. Perusal of the literature suggests that many workers do not provide evidence about which fate meets *Leishmania* under their experimental infections, which may contribute occasionally to seemingly contradictory results.

Experimental studies have shown that similar to *Leishmania*, treatment of macrophages with *Leishmania *glycoconjugates can likewise regulate the activation of NOS2 and production of NO. LPG can synergize with IFN-*γ* for the induction of NO expression in murine macrophages *in vitro*. However, incubation of macrophages with LPG-derived PG before stimulation with LPG plus IFN-*γ* led to inhibition of NOS2 expression [65]. These studies provided evidence that the interaction between the macrophage and the parasite impairs the activation of the microbicidal mechanisms of macrophages after exposure to IFN-*γ* in a process that is replicated by PG treatments. Given these findings, it was surprising that despite the complete absence of LPG or all PGs in the *lpg*1^−^ or *lpg*2^−^ mutants (described further below), mutant parasites remained “stealthy” and able to down regulate host cell activation [27, 66]. A similar contradiction was seen in studies of the smaller GIPL, which are highly abundant in both parasites stages and had been shown to inhibit NO synthesis by macrophage in a dose- and time-dependent manner, impairing its leishmanicidal activity [67]. However, mutants defective in the synthesis of the ether lipid anchor and thus lacking both LPG and GIPLs resembled the *lpg*1^−^ and *lpg*2^−^ mutants in remaining “stealthy” and inhibiting host cell activation [68]. This apparent paradox has not been resolved and has led to proposals that avoidance of host cell activation may be highly redundant amongst many parasite surface molecules, perhaps through their ability to interact with secondary ligands/mediators such as complement or other serum proteins. One attractive model is that macrophage deactivation is independent of surface molecules, instead depending on other processes such as secretion of parasite molecules through an exosomes-like or other pathways (reviewed in [69]). 

In addition to NO, activated macrophages employ other antimicrobial molecules, such as ROS or antimicrobial peptides, to kill intracellular parasites. ROS such as superoxide, hydrogen peroxide, and hydroxyl radicals, are produced after activation of NADPH oxidase and interact with pathogen phospholipid membranes, inducing damage and dead of pathogens [70]. Some evidence highlights the importance of ROS in control of *Leishmania* growth [71, 72]. Upon infection by *L. donovani *promastigotes, peritoneal macrophages elicit a strong respiratory burst with release of superoxide anion, thus favoring the elimination of the intracellular amastigotes. When infected in the presence of catalase, an enzyme that catalyze the decomposition of hydrogen peroxide to water and oxygen, macrophages lost their ability to kill *L. donovani *[73]. These results show that ROS are central compounds that act to eliminate intracellular *Leishmania in vitro*.

Respiratory burst activity and NO production are regulated by phosphorylation events mediated by protein kinase C (PKC) [74]. Infection with *Leishmania* is able to inhibit PKC activity in macrophages and several findings suggesting that LPG is related to this activity, thereby favoring intracellular survival of the parasite through inhibition of both oxidative burst and NO production [10, 75–79]. Besides PKC, production of cytokines such as IL-12 was inhibited in bone marrow-derived macrophages after infection with *L. major *[80]. Furthermore, purified LPG plays similar inhibitory effect over IL-12 production, probably thought the activation of the mitogen-activated protein kinase (MAPK) Erk 1/2, which suppresses IL-12 gene transcription [81]. Besides IL-12, purified LPG also suppressed IL-1*β* gene expression in THP-1 monocytes induced by endotoxin, TNF-*α* or *Staphylococcus* stimulation [82].

## 7. Recognition of LPG by Mammalian Innate Immune Receptors

The initiation of immune response against invading pathogens starts upon the interaction of microbial molecules with receptors of innate immune cells. Glycoconjugates expressed by protozoans interact with macrophage receptors and are recognized as foreign by immune system. Purified GPI-anchored surface proteins of *Plasmodium falciparum*, *Trypanosoma brucei* and *L. mexicana*, initiate the rapid activation of macrophage protein tyrosine kinases (PTKs) [83–85]. GPI anchors expressed by protozoans, such as *Plasmodium* and *Trypanosoma*, can activate the secretion of cytokines, such as IL-12 and TNF-*α*, and NO synthesis by macrophages [83, 85–90].

The activation of Toll-like receptors (TLRs) by microbial ligands recruits the adaptor protein MyD88 (myeloid differentiation primary response gene 88) and triggers intracellular signaling events, culminating on the activation of the transcription factor NF-*κ*B and its translocation to nucleus. NF-*κ*B in turn induces innate immune mechanisms such as the production of reactive oxygen and nitrogen intermediates, chemokine/cytokine secretion, and cellular differentiation [91]. Several evidences have suggested that *Leishmania* expresses ligands able to stimulate the TLRs signaling pathways. RAW macrophages selectively upregulated the IL-1*α* mRNA expression in response to *L. major* infection, and this was not observed when macrophages were transfected with a dominant-negative of MyD88 or when peritoneal macrophages derived from MyD88-deficient mice were infected with *L. major *[92]. In addition, mice deficient for MyD88 infected with *L. major* showed an increase in lesion size compared to their littermate controls [93]. These results suggest that *L. major* may express ligands for TLR activation. Accordingly, *L. major* LPG activated NF-*κ*B, the secretion of Th1-type cytokines, ROS, and NO by either human or murine macrophages in a mechanism dependent of TLR2 [93–95]. In addition, purified LPG upregulates TLR2 expression and stimulate IFN-*γ* and TNF-*α* secretion by human NK cells in a TLR2-dependent manner [96]. Thus, activation of TLR2 may contribute to host resistance against *Leishmania* and LPG is proposed to be a putative agonist for TLR activation.

In addition to macrophages and NK cells, LPG has been shown to exert stimulatory effects on dendritic cells (DCs). Purified *L. mexicana *LPG was able to induce the expression of CD86 and major histocompatibility complex class II (MHC-II) by DCs; furthermore, *L. major *LPG stimulated the expression of CD25, CD31, and vascular-endothelial cadherin by mouse Langerhans cells, albeit accompanied by inhibition of their migratory activity [97, 98]. Importantly, upregulation of stimulatory and costimulatory molecules in DCs occurs in response to activation of pattern recognition receptors; therefore, these studies corroborate the hypothesis that that *Leishmania* LPG triggers activation of these receptors.

Given the interaction of LPG with TLRs in the context of activated macrophages where this leads to a proinflammatory response and parasite control, an important but as yet unanswered question is how the LPG-TLR interaction fails to control parasite infection in unactivated macrophages. A variety of pathways are known which negatively regulate TLR signaling, and potentially one of these acts to mitigate TLR activation. A second question is whether LPG or related molecules are internalized into host cells, which would then place them in contact with variety internal sensors including the NOD-like receptors protein family in the cytosol. Early studies showed LPG trafficking into the interior of host cells [99] and recently several groups have provided evidence suggesting that *Leishmania* molecules may gain access to the host cytosol through some routes, potentially including an exosome-like pathway [100, 101]. Further work is needed to confirm these provocative hypotheses and explore the role of LPG and related glycoconjugates in this process.

## 8. The Assessment of LPG Functions by Using LPG-Defective Mutants

As mentioned above, many studies have used purified LPG, fragments thereof, or related molecules, to investigate their role in *Leishmania *pathogenesis and host response. However, LPG preparations can include contaminating molecules including proteins, unless proper precautions are taken, and use of exogenous LPG may not properly mimic the physiological location and concentrations of LPG delivered by infecting parasites. Moreover, as noted above, many LPG domains are shared by other parasite molecules, raising the possibility that functions attributed to LPG *in vitro* may actually be fulfilled by LPG-related molecules *in vivo*.

In the last few years, the generation of LPG mutants of *Leishmania* has provided powerful tools to identify the function of these molecules [102–105]. Of note, the recent identification of genes related to LPG biosynthetic pathways allowed the generation of “clean” LPG mutant strains by specific gene targeting. *Leishmania* are typically diploid although recent studies suggest that many chromosomes may be aneuploid, at least transiently [106]. Thus, two or more successive rounds of gene replacement are required to generate full homozygous null mutants, as while feasible in some cases sexual crossing remains challenging [107]. Importantly, the phenotypes of the mutants chosen for biological studies were rescued by complementation of the specific LPG gene into the parasite [108–110]. This rules out the well-known problem of loss of virulence during transfection or culture of *Leishmania*, which occurs sporadically in all species. Thus far nearly 20 genes affecting various steps of LPG biosynthesis have been described through complementation of LPG mutants or through various reverse genetic strategies.

For the study of virulence, this repertoire of LPG genes has enabled researchers to concentrate on key mutants that cleanly affect LPG or related molecules. The first genetic assessment of the role of LPG in parasite virulence and host immunity followed the identification of the *LPG1 *gene, which was recovered following complementation of the LPG-deficient R2D2 mutant of *L. donovani *[110]. This gene encodes a putative galactofuranosyl transferase involved in biosynthesis of the LPG glycan core, but not other galactofuranosyl-containing glycoconjugates whose synthesis depends on other LPG1-related transferases [111]. *lpg*1^−^ mutants of *L. major* or *L. donovani *do not express LPG on their surface, while the expression of other glycoconjugates remains normal [112, 113], rendering these ideal for studies of the biological roles mediated exclusively by LPG. *lpg*1^−^ mutants are highly susceptible to lysis by complement; sensitive to oxidative stress, and they fail to even transiently inhibit phagolysosomal fusion immediately after invasion [27]. Moreover, *L. major* *lpg*1^−^ showed an impaired ability to survive inside macrophages [27, 113] and in mouse infections were highly attenuated, as represented by an extreme delay in lesion progression [27, 113].

Interestingly, the generality of the role of LPG or even PGs in parasite survival in all *Leishmania* has been questioned based on similar genetic studies in *L. mexicana*, where a proper *lpg*1^−^ line shows no decrease in infectivity tests in macrophages or mice [112], although it is complement sensitive [114]. Despite these observations, the *lpg*1^−^ *L. mexicana *nonetheless showed some alterations in host response, with a poor ability to stimulate the expression of costimulatory molecules on mouse DCs, and it was found that *lpg*1^−^ *L. mexicana*-infected mice showed lower numbers of activated DCs in draining lymph nodes and were unable to control early parasite burden [97]. Thus, it appears that LPG plays a quantitatively or qualitatively different role in *L. mexicana* virulence, especially in directing the immune response. A similar contrast was found in studies of an *L. mexicana* *lpg*2^−^, discussed below [115]. Amongst many potential explanations, the architecture of the PV has been proposed to be a factor, as it exists as a “spacious, multiparasite” compartment in *L. mexicana* infections versus a “tight, uniparasitic” compartment in *L. major* and *L. donovani* [114]. Thus, the roles of LPG appear to differ both quantitatively and qualitative amongst species.

While the LPG-deficient *lpg*1^−^ parasites show severe attenuation in both *L. donovani *and *L. major,* studies in the latter species show that some parasites survive and go on to generate normal amastigotes, in keeping with the downregulation of LPG during development. Since other PG-containing glycoconjugates such as PPG are found throughout the life cycle, the role of the PG moieties generally was investigated by the use of a mutant globally affecting PGs. The *LPG2 *gene was identified by complementation of the* L. donovani C3PO *mutant [109] and was shown in a series of seminal studies in Turco's laboratory to encode the Golgi GDP-mannose transporter [116–118], one of the founding members of what is now known to be a large family of nucleoside sugar transporters [119]. LPG2 was also the first multispecific nucleotide sugar transporters to be described, being able to carry both GDP-D-Arabinopyranose and GDP-Fucose in addition to GDP-Man [116]. As noted earlier, *L. major* utilizes D-Arabinopyranose as an LPG side chain “capping” sugar, but neither a role nor glycoconjugates bearing fucose has been described in *Leishmania*, although low levels of GDP-Fuc have been observed in promastigotes [120].

*lp* *g*2^−^ mutant parasites lack all PGs, including LPG and PPG, but synthesize normal levels of GIPLs and gp63 [66]. *L. major* and *L. donovanI* *lpg*2^−^ mutants failed to survive in the midgut of sand fly vector and were unable to establish infection in macrophages. In animal infections, *L. major* parasites showed “persistence without pathology,” with parasites persisting at low levels for the life of infected animals—a situation reminiscent of the life-long infection following healing of *Leishmania* infections in experimental animals and humans [16, 66, 114]. This parallel was further extended by the demonstration that as in healed animals, *L. major* *lpg*2^−^ induced long-term immunity against challenge with a virulent strain of *L. major *[121]. Observations that lymphocytes isolated from *L. major* *lpg*2^−^-infected mice produced less IL-4 and IL-10 after stimulation *in vitro*, compared to cells isolated from *L. major* WT-infected mice, provided evidences about the anti-inflammatory properties of PGs over immune cells [122]. Importantly, similar effects on cytokine expression were seen in the *lpg*5A^−^/*lpg*5B^−^ double mutant, which also lacks all PGs, but through inactivation of Golgi UDP-Gal transporter activity [122, 123]. However, the *lpg*5A^−^/*lpg*5B^−^ mutant shows a virulence defect comparable to that of the *lpg*1^−^ rather than *lpg*2^−^ mutant [123].This suggests that the “persistence without pathology” phenotype of the *lpg*2^−^ mutant may arise from effects on gylcoconjugates other than PGs [123]. Thus, comparison amongst the well-characterized collection of LPG/PG mutants provides a “genetic sieve”, allowing assignment of the roles of LPG and PGs separately and in immune interaction from their roles in general parasite infectivity. These studies using *lpg*2^−^ mutant parasites provided evidence that PGs, in addition to LPG, play important roles in *Leishmania* virulence [122, 123].

## 9. Concluding Remarks

LPG is a key molecule mediating many important steps essential for *Leishmania *virulence, in the hostile environment of the sand fly vector midgut, or in the mammalian host. The identification of genes related to LPG synthesis allowed the generation of *Leishmania *strains defective in LPG. The uses of these mutants have provided valuable clues about the role of this glycoconjugate in the biology of *Leishmania*. We envisage that further studies using these and new mutants may elucidate important issues related to innate immune recognition and host cell activation by protozoan parasites. This information will greatly increase our understanding of both* Leishmania* pathogenesis and the recognition of protozoan parasites by the mammalian innate immune system.

## References

[1] WHO Control of the leishmaniasis.

[2] Kaye P, Scott P (2011). Leishmaniasis: complexity at the host-pathogen interface. *Nature Reviews Microbiology*.

[3] Antoine JC, Prina E, Courret N, Lang T (2004). Leishmania spp.: on the interactions they establish with antigen-presenting cells of their mammalian hosts. *Advances in Parasitology*.

[4] Button LL, McMaster WR (1988). Molecular cloning of the major surface antigen of Leishmania. *Journal of Experimental Medicine*.

[5] Elhay MJ, McConville MJ, Handman E (1988). Immunochemical characterization of a glyco-inositol-phospholipid membrane antigen of Leishmania major. *Journal of Immunology*.

[6] Etges R, Bouvier J, Bordier C (1986). The major surface protein of Leishmania promastigotes is a protease. *Journal of Biological Chemistry*.

[7] McConville MJ, Ferguson MAJ (1993). The structure, biosynthesis and function of glycosylated phosphatidylinositols in the parasitic protozoa and higher eukaryotes. *Biochemical Journal*.

[8] McConville MJ, Mullin KA, Ilgoutz SC, Teasdale RD (2002). Secretory pathway of trypanosomatid parasites. *Microbiology and Molecular Biology Reviews*.

[9] Sacks D, Kamhawi S (2001). Molecular aspects of parasite-vector and vector-host interactions in Leishmaniasis. *Annual Review of Microbiology*.

[10] Turco SJ, Sacks DL (1991). Expression of a stage-specific lipophosphoglycan in Leishmania major amastigotes. *Molecular and Biochemical Parasitology*.

[11] Ilg T, Handman E, Stierhof YD (1999). Proteophosphoglycans from Leishmania promastigotes and amastigotes. *Biochemical Society Transactions*.

[12] Turco SJ, Späth GF, Beverley SM (2001). Is lipophosphoglycan a virulence factor? A surprising diversity between Leishmania species. *Trends in Parasitology*.

[13] Schlein Y, Schnur LF, Jacobson RL (1990). Released glycoconjugate of indigenous Leishmania major enhances survival of a foreign L. major in Phlebotomus papatasi. *Transactions of the Royal Society of Tropical Medicine and Hygiene*.

[14] Ilg T (2001). Lipophosphoglycan of the protozoan parasite Leishmania: stage- and species-specific importance for colonization of the sandfly vector, transmission and virulence to mammals. *Medical Microbiology and Immunology*.

[15] Pimenta PFP, Turco SJ, McConville MJ, Lawyer PG, Perkins PV, Sacks DL (1992). Stage-specific adhesion of Leishmania promastigotes to the sandfly midgut. *Science*.

[16] Sacks DL, Modi G, Rowton E (2000). The role of phosphoglycans in Leishmania-sand fly interactions. *Proceedings of the National Academy of Sciences of the United States of America*.

[17] Kamhawi S, Ramalho-Ortigao M, Van MP (2004). A role for insect galectins in parasite survival. *Cell*.

[18] Myskova J, Svobodova M, Beverley SM, Volf P (2007). A lipophosphoglycan-independent development of Leishmania in permissive sand flies. *Microbes and Infection*.

[19] Svárovská A, Ant TH, Seblová V, Jecná L, Beverley SM, Volf P (2010). Leishmania major glycosylation mutants require phosphoglycans (lpg2-) but not lipophosphoglycan (lpg1-) for survival in permissive sand fly vectors. *PLoS Neglected Tropical Diseases*.

[20] Svobodová M, Bates PA, Volf P (1997). Detection of lectin activity in Leishmania promastigotes and amastigotes. *Acta Tropica*.

[21] Svobodova M, Volf P, Killick-Kendrick R (1996). Agglutination of Leishmania promastigotes by midgut lectins from various species of phlebotomine sandflies. *Annals of Tropical Medicine and Parasitology*.

[22] Beverley SM, Dobson DE (2004). Flypaper for parasites. *Cell*.

[23] Pimenta PFP, Saraiva EMB, Rowton E (1994). Evidence that the vectorial competence of phlebotomine sand flies for different species of Leishmania is controlled by structural polymorphisms in the surface lipophosphoglycan. *Proceedings of the National Academy of Sciences of the United States of America*.

[24] Rogers ME, Chance ML, Bates PA (2002). The role of promastigote secretory gel in the origin and transmission of the infective stage of Leishmania mexicana by the sandfly Lutzomyia longipalpis. *Parasitology*.

[25] Stierhof YD, Bates PA, Jacobson RL (1999). Filamentous proteophosphoglycan secreted by Leishmania promastigotes forms gel like three-dimensional networks that obstruct the digestive tract of infected sandfly vectors. *European Journal of Cell Biology*.

[26] Puentes SM, Da Silva RP, Sacks DL, Hammer CH, Joiner KA (1990). Serum resistance of metacyclic stage Leishmania major promastigotes is due to release of C5b-9. *Journal of Immunology*.

[27] Späth GF, Garraway LA, Turco SJ, Beverley SM (2003). The role(s) of lipophosphoglycan (LPG) in the establishment of Leishmania major infections in mammalian hosts. *Proceedings of the National Academy of Sciences of the United States of America*.

[28] Puentes SM, Dwyer DM, Bates PA, Joiner KA (1989). Binding and release of C3 from Leishmania donovani promastigotes during incubation in normal human serum. *Journal of Immunology*.

[29] Da Silva RP, Hall BF, Joiner KA, Sacks DL (1989). CR1, the C3b receptor, mediates binding of infective Leishmania major metacyclic promastigotes to human macrophages. *Journal of Immunology*.

[30] Mosser DM, Edelson PJ (1984). Activation of the alternative complement pathway by leishmania promastigotes: parasite lysis and attachment to macrophages. *Journal of Immunology*.

[31] Mosser DM, Edelson PJ (1985). The mouse macrophage receptor for C3bi (CR3) is a major mechanism in the phagocytosis of Leishmania promastigotes. *Journal of Immunology*.

[32] Mosser DM, Edelson PJ (1987). The third component of complement (C3) is responsible for the intracellular survival of Leishmania major. *Nature*.

[33] Mosser DM, Springer TA, Diamond MS (1992). Leishmania promastigotes require opsonic complement to bind to the human leukocyte integrin Mac-1 (CD11b/CD18). *Journal of Cell Biology*.

[34] Russell DG, Wright SD (1988). Complement receptor type 3 (CR3) binds to an Arg-Gly-Asp-containing region of the major surface glycoprotein, gp63, of Leishmania promastigotes. *Journal of Experimental Medicine*.

[35] Wilson ME, Pearson RD (1988). Roles of CR3 and mannose receptors in the attachment and ingestion of Leishmania donovani by human mononuclear phagocytes. *Infection and Immunity*.

[36] Marth T, Kelsall BL (1997). Regulation of interleukin-12 by complement receptor 3 signaling. *Journal of Experimental Medicine*.

[37] Sutterwala FS, Noel GJ, Clynes R, Mosser DM (1997). Selective suppression of interleukin-12 induction after macrophage receptor ligation. *Journal of Experimental Medicine*.

[38] Wright SD, Silverstein SC (1983). Receptors for C3b and C3bi promote phagocytosis but not the release of toxic oxygen from human phagocytes. *Journal of Experimental Medicine*.

[39] Carter CR, Whitcomb JP, Campbell JA, Mukbel RM, McDowell MA (2009). Complement receptor 3 deficiency influences lesion progression during Leishmania major infection in BALB/c Mice. *Infection and Immunity*.

[40] Stafford JL, Neumann NF, Belosevic M (2002). Macrophage-mediated innate host defense against protozoan parasites. *Critical Reviews in Microbiology*.

[41] Wilson ME, Pearson RD (1986). Evidence that Leishmania donovani utilizes a mannose receptor on human mononuclear phagocytes to establish intracellular parasitism. *Journal of Immunology*.

[42] Green PJ, Feizi T, Stoll MS, Thiel S, Prescott A, McConville MJ (1994). Recognition of the major cell surface glycoconjugates of Leishmania parasites by the human serum mannan-binding protein. *Molecular and Biochemical Parasitology*.

[43] Culley FJ, Harris RA, Kaye PM, McAdam KPWJ, Raynes JG (1996). C-reactive protein binds to a novel ligand on Leishmania donovani and increases uptake into human macrophages. *Journal of Immunology*.

[44] Ballou SP, Lozanski G (1992). Induction of inflammatory cytokine release from cultured human monocytes by C-reactive protein. *Cytokine*.

[45] Galve-de Rochemonteix B, Wiktorowicz K, Kushner I, Dayer JM (1993). C-reactive protein increases production of IL-1*α*, IL-1*β*, and TNF-*α*, and expression of mRNA by human alveolar macrophages. *Journal of Leukocyte Biology*.

[46] Bodman-Smith KB, Mbuchi M, Culley FJ, Bates PA, Raynes JG (2002). C-reactive protein-mediated phagocytosis of Leishmania donovani promastigotes does not alter parasite survival or macrophage responses. *Parasite Immunology*.

[47] Desjardins M (1995). Biogenesis of phagolysosomes: the ’kiss and run’ hypothesis. *Trends in Cell Biology*.

[48] Desjardins M, Huber LA, Parton RG, Griffiths G (1994). Biogenesis of phagolysosomes proceeds through a sequential series of interactions with the endocytic apparatus. *Journal of Cell Biology*.

[49] Descoteaux A, Turco SJ (1999). Glycoconjugates in Leishmania infectivity. *Biochimica et Biophysica Acta*.

[50] Desjardins M, Descoteaux A (1997). Inhibition of phagolysosomal biogenesis by the Leishmania lipophosphoglycan. *Journal of Experimental Medicine*.

[51] Lodge R, Diallo TO, Descoteaux A (2006). Leishmania donovani lipophosphoglycan blocks NADPH oxidase assembly at the phagosome membrane. *Cellular Microbiology*.

[52] Chan J, Fujiwara T, Brennan P (1989). Microbial glycolipids: possible virulence factors that scavenge oxygen radicals. *Proceedings of the National Academy of Sciences of the United States of America*.

[53] Bahr V, Stierhof YD, Ilg T, Demar M, Quinten M, Overath P (1993). Expression of lipophosphoglycan, high-molecular weight phosphoglycan and glycoprotein 63 in promastigotes and amastigotes of Leishmania mexicana. *Molecular and Biochemical Parasitology*.

[54] McConville MJ, Blackwell JM (1991). Developmental changes in the glycosylated phosphatidylinositols of Leishmania donovani. *Journal of Biological Chemistry*.

[55] MacMicking J, Xie QW, Nathan C (1997). Nitric oxide and macrophage function. *Annual Review of Immunology*.

[56] Dong Z, Qi X, Xie K, Fidler IJ (1993). Protein tyrosine kinase inhibitors decrease induction of nitric oxide synthase activity in lipopolysaccharide-responsive and lipopolysaccharide-nonresponsive murine macrophages. *Journal of Immunology*.

[57] Stenger S, Thüring H, Röllinghoff M, Bogdan C (1994). Tissue expression of inducible nitric oxide synthase is closely associated with resistance to Leishmania major. *Journal of Experimental Medicine*.

[58] Mukbel RM, Patten C, Gibson K, Ghosh M, Petersen C, Jones DE (2007). Macrophage killing of Leishmania amazonensis amastigotes requires both nitric oxide and superoxide. *American Journal of Tropical Medicine and Hygiene*.

[59] Murray HW, Nathan F (1999). Macrophage microbicidal mechanisms in vivo: reactive nitrogen versus oxygen intermediates in the killing of intracellular visceral Leishmania donovani. *Journal of Experimental Medicine*.

[60] Wei XQ, Charles IG, Smith A (1995). Altered immune responses in mice lacking inducible nitric oxide synthase. *Nature*.

[61] Diefenbach A, Schindler H, Donhauser N (1998). Type 1 interferon (IFN*α*/*β*) and type 2 nitric oxide synthase regulate the innate immune response to a protozoan parasite. *Immunity*.

[62] Liew FY, Millott S, Parkinson C, Palmer RMJ, Moncada S (1990). Macrophage killing of Leishmania parasite in vivo is mediated by nitric oxide from L-arginine. *Journal of Immunology*.

[63] Stenger S, Donhauser N, Thüring H, Röllinghoff M, Bogdan C (1996). Reactivation of latent leishmaniasis by inhibition of inducible nitric oxide synthase. *Journal of Experimental Medicine*.

[64] Reiner SL, Zheng S, Wang ZE, Stowring L, Locksley RM (1994). Leishmania promastigotes evade interleukin 12 (IL-12) induction by macrophages and stimulate a broad range of cytokines from CD4^+^ T cells during initiation of infection. *Journal of Experimental Medicine*.

[65] Proudfoot L, Nikolaev AV, Feng GJ (1996). Regulation of the expression of nitric oxide synthase and leishmanicidal activity by glycoconjugates of Leishmania lipophosphoglycan in murine macrophages. *Proceedings of the National Academy of Sciences of the United States of America*.

[66] Späth GF, Lye LF, Segawa H, Sacks DL, Turco SJ, Beverley SM (2003). Persistence without pathology in phosphoglycan-deficient Leishmania major. *Science*.

[67] Proudfoot L, O’Donnell CA, Liew FY (1995). Glycoinositolphospholipids of Leishmania major inhibit nitric oxide synthesis and reduce leishmanicidal activity in murine macrophages. *European Journal of Immunology*.

[68] Zufferey R, Allen S, Barron T (2003). Ether phospholipids and glycosylinositolphospholipids are not required for amastigote virulence or for inhibition of macrophage activation by Leishmania major. *Journal of Biological Chemistry*.

[69] Silverman JM, Reiner NE (2011). Exosomes and other microvesicles in infection biology: organelles with unanticipated phenotypes. *Cellular Microbiology*.

[70] Babior BM (1999). NADPH oxidase: an update. *Blood*.

[71] Blos M, Schleicher U, Soares Rocha FJ, Meißner U, Röllinghoff M, Bogdan C (2003). Organ-specific and stage-dependent control of Leishmania major infection by inducible nitric oxide synthase and phagocyte NADPH oxidase. *European Journal of Immunology*.

[72] Murray HW (1982). Cell-mediated immune response in experimental visceral leishmaniasis. II. Oxygen-dependent killing of intracellular Leishmania donovani amastigotes. *Journal of Immunology*.

[73] Haidaris CG, Bonventre PF (1982). A role for oxygen-dependent mechanisms in killing of Leishmania donovani tissue forms by activated macrophages. *Journal of Immunology*.

[74] Nishizuka Y (1988). The molecular heterogeneity of protein kinase C and its implications for cellular regulation. *Nature*.

[75] Bhattacharyya S, Ghosh S, Jhonson PL, Bhattacharya SK, Majumdar S (2001). Immunomodulatory role of interleukin-10 in visceral leishmaniasis: defective activation of protein kinase C-mediated signal transduction events. *Infection and Immunity*.

[76] Descoteaux A, Matlashewski G, Turco SJ (1992). Inhibition of macrophage protein kinase C-mediated protein phosphorylation by Leishmania donovani lipophosphoglycan. *Journal of Immunology*.

[77] Descoteaux A, Turco SJ (1993). The lipophosphoglycan of Leishmania and macrophage protein kinase C. *Parasitology Today*.

[78] Moore KJ, Labrecque S, Matlashewski G (1993). Alteration of Leishmania donovani infection levels by selective impairment of macrophage signal transduction. *Journal of Immunology*.

[79] Olivier M, Brownsey RW, Reiner NE (1992). Defective stimulus-response coupling in human monocytes infected with Leishmania donovani is associated with altered activation and translocation of protein kinase C. *Proceedings of the National Academy of Sciences of the United States of America*.

[80] Carrera L, Gazzinelli RT, Badolato R (1996). Leishmania promastigotes selectively inhibit interleukin 12 induction in bone marrow-derived macrophages from susceptible and resistant mice. *Journal of Experimental Medicine*.

[81] Feng GJ, Goodridge HS, Harnett MM (1999). Extracellular signal-related kinase (ERK) and p38 mitogen-activated protein (MAP) kinases differentially regulate the lipopolysaccharide-mediated induction of inducible nitric oxide synthase and IL-12 in macrophages: Leishmania phosphoglycans subvert macrophage IL-12 production by targeting ERK MAP kinase. *Journal of Immunology*.

[82] Hatzigeorgiou DE, Geng J, Zhu B (1996). Lipophosphoglycan from Leishmania suppresses agonist-induced interleukin 1*β* gene expression in human monocytes via a unique promoter sequence. *Proceedings of the National Academy of Sciences of the United States of America*.

[83] Ropert C, Gazzinelli RT (2000). Signaling of immune system cells by glycosylphosphatidylinositol (GPI) anchor and related structures derived from parasitic protozoa. *Current Opinion in Microbiology*.

[84] Tachado SD, Gerold P, Schwarz R, Novakovic S, Mcconville M, Schofield L (1997). Signal transduction in macrophages by glycosylphosphatidylinositols of Plasmodium, Trypanosoma, and Leishmania: activation of protein tyrosine kinases and protein kinase C by inositolglycan and diacylglycerol moieties. *Proceedings of the National Academy of Sciences of the United States of America*.

[85] Tachado SD, Schofield L (1994). Glycosylphosphatidylinositol toxin of Trypanosoma brucei regulates IL-1*α* and TNF-*α* expression in macrophages by protein tyrosine kinase mediated signal transduction. *Biochemical and Biophysical Research Communications*.

[86] Almeida IC, Camargo MM, Procópio DO (2000). Highly purified glycosylphosphatidylinositols from Trypanosoma cruzi are potent proinflammatory agents. *EMBO Journal*.

[87] Camargo MM, Almeida IC, Pereira MES, Ferguson MAJ, Travassos LR, Gazzinelli RT (1997). Glycosylphosphatidylinositol-anchored mucin-like glycoproteins isolated from Trypanosoma cruzi trypomastigotes initiate the synthesis of proinflammatory cytokines by macrophages. *Journal of Immunology*.

[88] Camargo MM, Andrade AC, Almeida IC, Travassos LR, Gazzinelli RT (1997). Glycoconjugates isolated from Trypanosoma cruzi but not from Leishmania species membranes trigger nitric oxide synthesis as well as microbicidal activity in IFN-*γ*-primed macrophages. *Journal of Immunology*.

[89] Magez S, Stijlemans B, Radwanska M, Pays E, Ferguson MAJ, De Baetselier P (1998). The glycosyl-inositol-phosphate and dimyristoylglycerol moieties of the glycosylphosphatidylinositol anchor of the trypanosome variant-specific surface glycoprotein are distinct macrophage-activating factors. *Journal of Immunology*.

[90] Tachado SD, Gerold P, McConville MJ (1996). Glycosylphosphatidylinositol toxin of Plasmodium induces nitric oxide synthase expression in macrophages and vascular endothelial cells by a protein tyrosine kinase-dependent and protein kinase C-dependent signaling pathway. *Journal of Immunology*.

[91] Akira S, Takeda K (2004). Toll-like receptor signalling. *Nature Reviews Immunology*.

[92] Hawn TR, Ozinsky A, Underhill DM, Buckner FS, Akira S, Aderem A (2002). Leishmania major activates IL-1*α* expression in macrophages through a MyD88-dependent pathway. *Microbes and Infection*.

[93] de Veer MJ, Curtis JM, Baldwin TM (2003). MyD88 is essential for clearance of Leishmania major: possible role for lipophosphoglycan and Toll-like receptor 2 signaling. *European Journal of Immunology*.

[94] Kavoosi G, Ardestani SK, Kariminia A (2009). The involvement of TLR2 in cytokine and reactive oxygen species (ROS) production by PBMCs in response to Leishmania major phosphoglycans (PGs). *Parasitology*.

[95] Kavoosi G, Ardestani SK, Kariminia A, Alimohammadian MH (2010). Leishmania major lipophosphoglycan: discrepancy in toll-like receptor signaling. *Experimental Parasitology*.

[96] Becker I, Salaiza N, Aguirre M (2003). Leishmania lipophosphoglycan (LPG) activates NK cells through toll-like receptor-2. *Molecular and Biochemical Parasitology*.

[97] Aebischer T, Bennett CL, Pelizzola M (2005). A critical role for lipophosphoglycan in proinflammatory responses of dendritic cells to Leishmania mexicana. *European Journal of Immunology*.

[98] Ponte-Sucre A, Heise D, Moll H (2001). Leishmania major lipophosphoglycan modulates the phenotype and inhibits migration of murine Langerhans cells. *Immunology*.

[99] Tolson DL, Turco SJ, Pearson TW (1990). Expression of a repeating phosphorylated disaccharide lipophosphoglycan epitope on the surface of macrophages infected with Leishmania donovani. *Infection and Immunity*.

[100] Silverman JM, Clos J, De’Oliveira CC (2010). An exosome-based secretion pathway is responsible for protein export from Leishmania and communication with macrophages. *Journal of Cell Science*.

[101] Silverman JM, Clos J, Horakova E (2010). Leishmania exosomes modulate innate and adaptive immune responses through effects on monocytes and dendritic cells. *Journal of Immunology*.

[102] Butcher BA, Turco SJ, Hilty BA, Pimentai PF, Panunzio M, Sacks DL (1996). Deficiency in *β*1,3-galactosyltransferase of a Leishmania major lipophosphoglycan mutant adversely influences the Leishmania-sand fly interaction. *Journal of Biological Chemistry*.

[103] Descoteaux A, Mengeling BJ, Beverley SM, Turco SJ (1998). Leishmania donovani has distinct mannosylphosphoryltransferases for the initiation and elongation phases of lipophosphoglycan repeating unit biosynthesis. *Molecular and Biochemical Parasitology*.

[104] King DL, Turco SJ (1988). A ricin agglutinin-resistant clone of Leishmania donovani deficient in lipophosphoglycan. *Molecular and Biochemical Parasitology*.

[105] McNeeley TB, Tolson DL, Pearson TW, Turco SJ (1990). Characterization of Leishmania donovani variant clones using anti-lipophosphoglycan monoclonal antibodies. *Glycobiology*.

[106] Sterkers Y, Lachaud L, Crobu L, Bastien P, Pagès M (2011). FISH analysis reveals aneuploidy and continual generation of chromosomal mosaicism in Leishmania major. *Cellular Microbiology*.

[107] Akopyants NS, Kimblin N, Secundino N (2009). Demonstration of genetic exchange during cyclical development of Leishmania in the sand fly vector. *Science*.

[108] Beverley SM, Turco SJ (1998). Lipophosphoglycan (LPG) and the identification of virulence genes in the protozoan parasite Leishmania. *Trends in Microbiology*.

[109] Descoteaux A, Luo Y, Turco SJ, Beverley SM (1995). A specialized pathway affecting virulence glycoconjugates of Leishmania. *Science*.

[110] Ryan KA, Garraway LA, Descoteaux A, Turco SJ, Beverley SM (1993). Isolation of virulence genes directing surface glycosyl-phosphatidylinositol synthesis by functional complementation of Leishmania. *Proceedings of the National Academy of Sciences of the United States of America*.

[111] Zhang K, Barron T, Turco SJ, Beverley SM (2004). The LPG1 gene family of Leishmania major. *Molecular and Biochemical Parasitology*.

[112] Ilg T (2000). Lipophosphoglycan is not required for infection of macrophages or mice by Leishmania mexicana. *EMBO Journal*.

[113] Späth GF, Epstein L, Leader B (2000). Lipophosphoglycan is a virulence factor distinct from related glycoconjugates in the protozoan parasite Leishmania major. *Proceedings of the National Academy of Sciences of the United States of America*.

[114] Gaur U, Showalter M, Hickerson S (2009). Leishmania donovani lacking the Golgi GDP-Man transporter LPG2 exhibit attenuated virulence in mammalian hosts. *Experimental Parasitology*.

[115] Ilg T, Demar M, Harbecke D (2001). Phosphoglycan repeat-deficient Leishmania mexicana parasites remain infectious to macrophages and mice. *Journal of Biological Chemistry*.

[116] Hong K, Ma D, Beverley SM, Turco SJ (2000). The Leishmania GDP-mannose transporter is an autonomous, multi-specific, hexameric complex of LPG2 subunits. *Biochemistry*.

[117] Ma D, Russell DG, Beverley SM, Turco SJ (1997). Golgi GDP-mannose uptake requires leishmania LPG2: a member of a eukaryotic family of putative nucleotide-sugar transporters. *Journal of Biological Chemistry*.

[118] Segawa H, Soares RP, Kawakita M, Beverley SM, Turco SJ (2005). Reconstitution of GDP-mannose transport activity with purified Leishmania LPG2 protein in liposomes. *Journal of Biological Chemistry*.

[119] Caffaro CE, Hirschberg CB (2006). Nucleotide sugar transporters of the Golgi apparatus: from basic science to diseases. *Accounts of Chemical Research*.

[120] Turnock DC, Ferguson MAJ (2007). Sugar nucleotide pools of Trypanosoma brucei, Trypanosoma cruzi, and Leishmania major. *Eukaryotic Cell*.

[121] Uzonna JE, Späth GF, Beverley SM, Scott P (2004). Vaccination with phosphoglycan-deficient Leishmania major protects highly susceptible mice from virulent challenge without inducing a strong Th1 response. *Journal of Immunology*.

[122] Liu D, Kebaier C, Pakpour N (2009). Leishmania major phosphoglycans influence the host early immune response by modulating dendritic cell functions. *Infection and Immunity*.

[123] Capul AA, Hickerson S, Barron T, Turco SJ, Beverley SM (2007). Comparisons of mutants lacking the golgi UDP-galactose or GDP-mannose transporters establish that phosphoglycans are important for promastigote but not amastigote virulence in Leishmania major. *Infection and Immunity*.

